# The Clinical Utility of fMRI for Identifying Covert Awareness in the Vegetative State: A Comparison of Sensitivity between 3T and 1.5T

**DOI:** 10.1371/journal.pone.0095082

**Published:** 2014-04-14

**Authors:** Davinia Fernández-Espejo, Loretta Norton, Adrian M. Owen

**Affiliations:** 1 The Brain and Mind Institute, The University of Western Ontario, London, Ontario, Canada; 2 Department of Neurocritical Care, University of Western Ontario, London Health Sciences Centre-University Hospital, London, Ontario, Canada; Penn State University, United States of America

## Abstract

In the last few years, mental imagery fMRI paradigms have been used successfully to identify covert command-following and awareness in some patients who are thought to be entirely vegetative. However, to date there is only evidence supporting their use at magnetic fields of 3T, which limits their applicability in clinical settings where lower field strengths are typically used. Here, we test the ‘gold standard’ fMRI paradigm for detecting residual awareness in non-responsive patients by comparing its sensitivity at 1.5T and 3T in the same group of healthy volunteers. We were able to successfully detect brain activity showing command-following in most participants at both 3T and 1.5T, with similar reliability. These results demonstrate that fMRI assessment of covert awareness is clinically viable and therefore justify a broader use of these methods in standard assessments in severely brain injured patients.

## Introduction

The vegetative state is a clinical condition that is often described as ‘wakefulness without awareness’ [1]. The presence of awareness is clinically measured by the ability to follow commands -either verbally, or behaviourally. However, recent advances in the field of functional neuroimaging, have demonstrated that behavioural assessment is not sufficient to fully capture the internal status of all vegetative state patients [2], [3]. Owen and colleagues [4] introduced a method for eliciting covert command-following with functional magnetic resonance imaging (fMRI) that involved asking participants to imagine hitting a tennis ball and to imagine walking from room to room in their house while in the scanner. Using this technique, a patient who fulfilled all of the internationally agreed clinical criteria for the vegetative state was shown to be covertly aware and able to wilfully respond to commands by simply modulating her brain activity [4]. These two mental imagery paradigms were later proven to be the most robust tasks for yielding reliable single subject activity in healthy volunteers [5] and, therefore, have become the gold-standard for assessing the presence of volition in non-communicative brain injured-patients [5], [6].

Using this fMRI method, and a similar approach based on EEG, it has been estimated that up to 20% of patients who are thought to be entirely vegetative even after careful and repeated standard behavioural testing [7] may be aware and capable of demonstrating command following when assessed with neuroimaging tools that do not require any overt behavioural output [8], [9]. Moreover, the same method has been used to successfully establish accurate functional communication in several non-communicative patients who clinically appeared to be in a vegetative state [2], [9].

It has been argued that the increasing evidence for covert awareness and communication in some non-responsive patients calls for a re-evaluation of the existing diagnostic categories and guidelines for behaviourally non-responsive patients and for the development and formal inclusion of validated, standardised neuroimaging procedures in those guidelines [2], [6], [10]. Before this is possible, however, its methodological plausibility needs to be tested. While all the evidence supporting the robustness and reliability of the fMRI methods to detect covert awareness comes from data collected at a field strength of 3T (the typical field strengths used in research applications), most clinical scanners use a lower field strength of 1.5 T [11] and to date, there is no clear evidence that these methods would generate reliable results at this field strength.

Here, we compared the activation elicited by the mental imagery paradigms most commonly used to detect covert awareness (i.e. motor imagery and spatial navigation) in a group of healthy volunteers at 3T (in an imaging research centre) and 1.5T (in a standard clinical setting), in order to assess their reliability for detecting single subject activations at lower fields for their potential clinical use.

## Material and Methods

### Ethics Statement

All volunteers gave written informed consent and were paid for their participation in the experiment. Ethical approval for the study was provided by the Health Sciences Research Ethics Board of the University of Western Ontario.

### Participants

Fifteen right-handed healthy volunteers (23±3 years, 9 males) took part in the study. None of the volunteers declared any history of neurological or psychiatric disease.

### Imagery tasks

While in the MRI scanner, all participants were asked to perform two mental imagery tasks, i.e. motor imagery and spatial navigation, as described elsewhere [4], [5], [9]. In the motor imagery task, participants were instructed to imagine swinging an arm to hit a tennis ball in a tennis match. In the spatial navigation task, they were instructed to imagine walking from room to room in their house and visualise all objects they would encounter if they were in their home. The subjects were asked to alternate 30-second periods of mental imagery with 30-second periods of rest for a total of 5:30 minutes. The beginning of each imaginary period was cued with the word ‘tennis’ or ‘house’ and the rest periods were cued with the word ‘relax’.

### Image acquisition

Data was acquired in a 3T Siemens scanner (Magnetom Trio Tim, Siemens, Germany) with a Siemens 32-channel head-coil at the Centre for Functional and Metabolic Mapping (CFMM) at Robarts Research Institute, and a 1.5 General Electric scanner (Signa Excite, GE, Fairfield, CT) at the University Hospital LHSC (London Health Sciences Centre). The order of the session was counterbalanced across participants. Fourteen participants underwent both scanning sessions (7 had the 3T session first) while one participant was only scanned in the 3T scanner and therefore discarded for the comparative analyses. Time between sessions ranged from 4 to 114 days (27±3).

The MRI protocol at 3T included a single session of 165 volumes, of 36 axial slices each covering the whole brain, using echo-planar images (TR = 2000 ms, TE  = 30 ms, matrix size  = 70×70, slice thickness  = 3 mm, in-plane resolution  = 3×3 mm, flip angle  = 78°). High-resolution T1-weighted 3D MP-rage images (TR = 2300 ms, TE = 2.98 ms, IT = 900, matrix size  = 256×240, voxel size 1×1×1 mm, flip angle = 9°) were acquired in the same session.

Two sessions of each mental imagery task were acquired at 1.5T as we were expecting less power due to the lower field strength. Each1.5T session included a T2*-weighted one-shot spiral-in containing 134 volumes of 30 slices (TR = 2500 ms, TE = 40 ms, matrix size = 64×64, slice thickness  = 5 mm, in plane resolution = 3.75×3.75 mm, flip angle  = 90°). A T1-weighted 3d-SPGR pulse sequence (TR = 9.2-10.2 ms, TE = 4 ms, matrix size = 256×256, voxel size = 1.02×1.02×1.40 mm, flip angle  = 10°, thickness = 1.4 mm, FOV = 24 cm, NEX = 1.0) was also acquired in the same session.

The task instructions and cues were presented using E-Prime® 2.0 running on Windows XP on an iMac computer and an MRI-compatible high-quality digital sound system incorporating noise-attenuated headphones (Silent Scan™, Avotec Inc.) at 3T. At 1.5T, we used SuperLab 4.0 running on a Windows XP PC and noise-attenuated MRI-compatible headphones (Resonance Technology Inc.).

### fMRI data analysis

Data was pre-processed and analysed using SPM8 (http://www.fil.ion.ucl.ac.uk/spm). Data was first manually AC-PC reoriented. Spatial pre-processing included: realignment to correct subjects' motion, co-registration between the structural and functional data sets, spatial normalising at the native resolution of the data acquired in the 1.5 scanner (for comparison reasons) and smoothing with an 8-mm FWHM Gaussian kernel.

Single subject fixed-effect analyses were performed in each subject. The analysis was based on the general linear model using the canonical hemodynamic response function [12]. Each scan was modelled to belong to the mental imagery (i.e. motor imagery or spatial navigation) or the rest condition. Movement parameters calculated from the realignment step were also included as covariates of non interest. High-pass filtering using a cut-off period of 128 seconds was implemented in order to remove slow-signal drifts from the time series. Linear contrasts were used to obtain subject-specific estimates of each of the effects of interest. The contrast images containing these estimates for each voxel were then smoothed at 8 mm FWHM Gausian kernel in order to increase inter-subject averaging at the group level, taking into account inter-individual anatomical variability. This linear increase in smoothing from first to second level improves statistical power at the group level while allowing spatially accurate results at the first level [5]. The smoothed contrast images were then entered into group analyses across the 14 participants who completed both sessions. One-sample t-tests were performed to obtain the patterns of activity for the 3T and 1.5T data. Paired t-tests were performed to test possible differences between the data obtained from the two centres. In all cases, the statistical threshold was set at a Family Wise Error (FWE) corrected p<0.05 on 10 mm spherical ROIs centered on previously documented coordinates: supplementary motor area (SMA), pre-SMA, dorsal premotor cortex and inferior parietal lobule for motor imagery; pre-SMA, dorsal premotor cortex, parahippocampal cortex, retrosplenial cortex, occipito-parietal junction and precuneus for spatial navigation [5]; see Experiments 1 and 2).

## Results

### Motor Imagery

**Figure 1:**
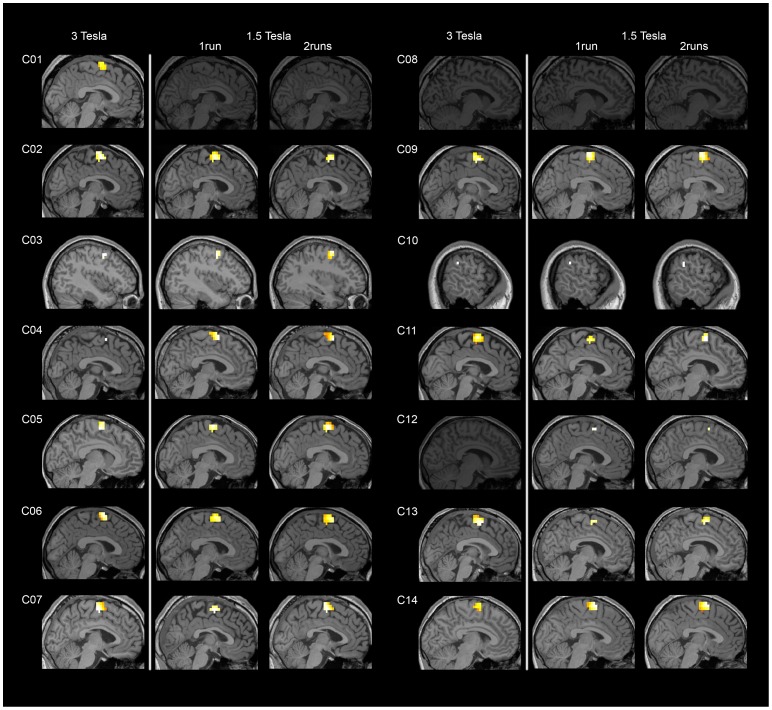
Example of individual results for the motor imagery task versus rest. For each subject, the ROI showing highest consistency across scanning sessions is displayed. All participants, except C03, C08 and C10, showed activation of SMA in at least one scanning session. Participants C03 and C10 activated other anatomically appropriate areas (dorsal premotor cortex and inferior parietal lobule respectively). Participant C08 failed to show any significant activation for any scanning session. Participant C01 failed to show any significant activation in the scanning session at 1.5T while participant C12 failed to do so at 3T. Results are thresholded at a FWE-corrected p<0.05.

**Figure 2:**
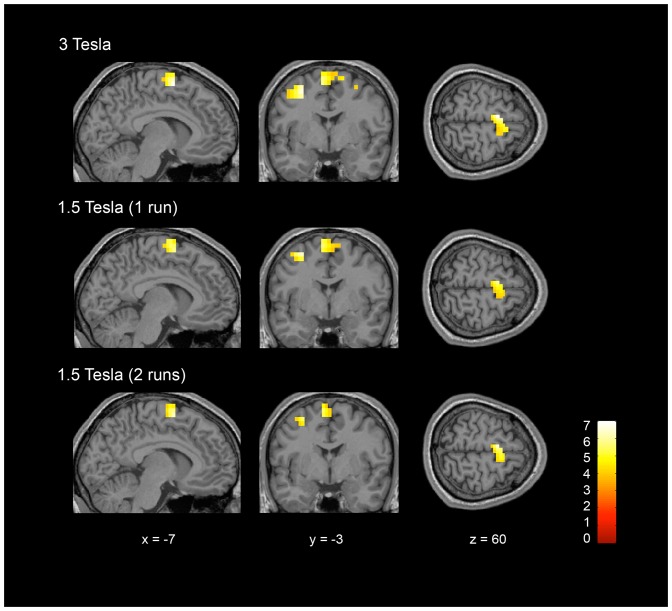
ROI group results showing activation of SMA and dorsal premotor cortex for the motor imagery task (i.e. imagine playing tennis) compared to rest at 3T and 1.5T (including 1 and 2 runs). For display, results are thresholded at an uncorrected p<0.001 and rendered on a canonical single subject T1 MRI image.

Motor imagery (imagine playing tennis) compared to rest elicited significant activity in all the studied ROIs (i.e. SMA, pre-SMA, dorsal premotor cortex and inferior parietal lobule) and for most of the healthy volunteers (see Table 1 and Figure 1), at both 3T and 1.5T. Group activations are shown in Table 2 and Figure 2.

**Table 1:** Individual results for the motor imagery (tennis) task.

Subject number	1	2	3	4	5	6	7	8	9	10	11	12	13	14	Total	Peak Coordinates
																(x,y,z; mean ± SD)
*1.5T (1 run)*																
SMA	−	+	−	+	+	+	+	−	+	−	+	+	+	+	10	−3±5, −2±4, 63±4
Pre-SMA	−	+	−	+	+	+	+	−	+	−	−	+	+	+	9	5±2, 3±4, 62±4
Dorsal Premotor Cortex	−	+	+	+	+	+	+	−	+	−	+	+	+	+	11	−32±5, −3±5, 55±2
																39±6, 2±5, 51±4
Inferior Parietal Lobule	−	+	−	+	+	+	−	−	+	+	−	+	+	+	9	−61±2, −40±3, 23±20
																54±5, −37±3, 32±7
Total	0	4	1	4	4	4	3	0	4	1	2	4	4	4		
*1.5T (2 runs)*																
SMA	−	+	+	+	+	+	+	−	+	−	+	+	+	+	11	−1±5, = 2±4, 62±4
Pre-SMA	−	+	−	+	+	+	+	−	+	+	−	+	+	+	10	4±5, 4±6, 62±3
Dorsal Premotor Cortex	−	+	+	+	+	+	+	−	+	+	+	+	+	+	12	−33±5, −3±4, 54±3
																39±7, −0±4, 52±4
Inferior Parietal Lobule	−	+	−	+	+	+	−	+	+	+	−	+	+	+	10	−60±2, −42±4, 20±22
																57±3, −37±4, 31±7
Total	0	4	2	4	4	4	3	1	4	3	2	4	4	4		
*3T*																
SMA	+	+	+	+	+	+	+	−	+	+	+	−	+	+	12	−2±4, −2±4, 63±3
Pre-SMA	+	+	−	+	+	+	+	−	+	+	+	−	+	+	11	5±15, 1±4, 63±5
Dorsal Premotor Cortex	+	+	+	+	+	+	+	−	+	+	+	−	+	+	12	−32±4, −6±3, 53±3
																42±6, 0±4, 52±5
Inferior Parietal Lobule	−	−	−	−	−	+	−	−	+	+	+	−	+	+	6	−60±2, −41±2, 32±3
																56±2, −36±4, 29±3
Total	3	3	2	3	3	4	3	0	4	4	4	0	4	4		

Results thresholded at FWE-corrected p<0.05 on the studied ROIs.

**Table 2:** Group results for the motor imagery (tennis) task.

Brain area	x	y	z	Z value	p value
*1.5 T 1 run*					
Pre-SMA	4	4	60	3.45	0.004
SMA	−7	0	60	3.94	0.001
Dorsal premotor cortex	−29	−3	55	4.15	0.000
*1.5 T 2 runs*					
Pre-SMA	4	4	60	3.74	0.002
SMA	−7	0	60	4.11	0.000
Dorsal premotor cortex	−29	−7	55	3.89	0.001
Inferior parietal lobule	−59	−41	35	2.67	0.031
*3T*					
Pre-SMA	1	4	65	3.78	0.002
SMA	−7	0	60	4.31	0.000
Dorsal premotor cortex	−29	−3	50	4.25	0.000
	34	−7	55	3.44	0.005

Regions showing significant activation versus rest for the 1.5T and 3T data. Results thresholded at FWE-corrected p<0.05.

### Spatial Navigation

**Figure 3:**
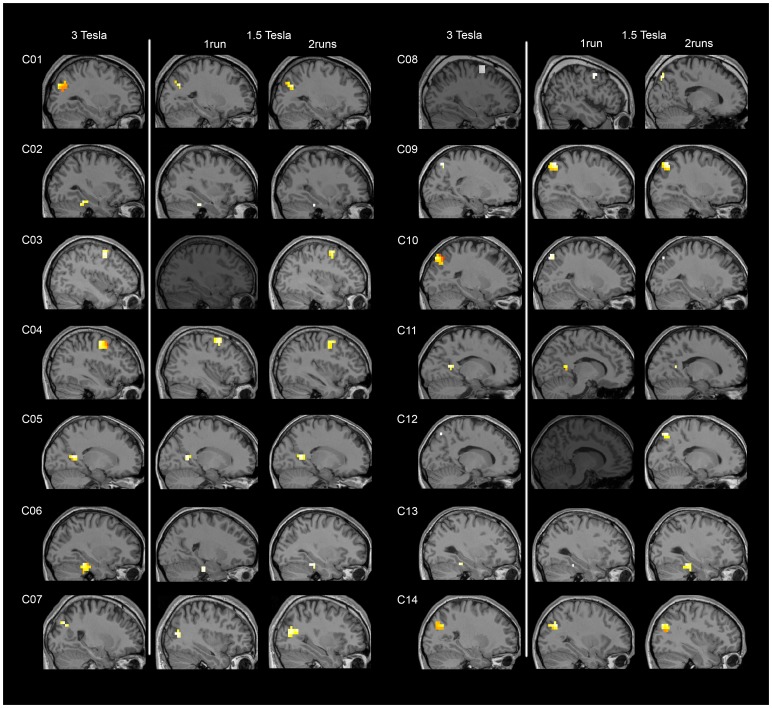
Example of individual results for the motor imagery task versus rest. For each subject, the ROI showing highest consistency across scanning sessions is displayed: occipito-parietal junction (C01, C07 C14), parahippocampal cortex (C02, C06, C13), dorsal premotor cortex (C03, C04, C08 at 3T and 1 run at 1.5T), retrosplenial cortex (C05, C11), and precuneus (C08 at 1.5T, C09, C10, C12). Participants C03 and C12 failed to show significant activation in the scanning session at 1.5T when only 1 run was analyzed but succeeded when both runs were analyzed. Results are thresholded at a FWE-corrected p<0.05.

**Figure 4:**
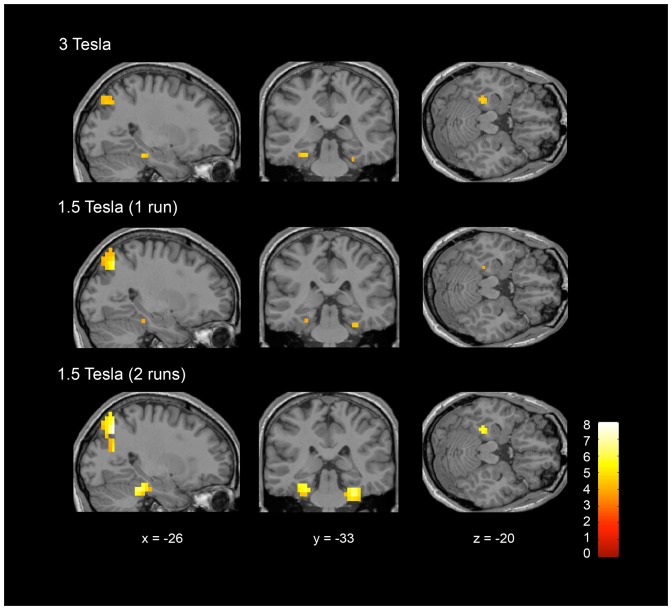
ROI group results showing activation of the parahippocampal cortex and the occipito-parietal junction for the spatial navigation task (i.e. imagine walking around your house) compared to rest at 3T and 1.5T (including 1 and 2 runs). For display, results are thresholded at an uncorrected p<0.001 and rendered on a canonical single subject T1 MRI image.

Spatial navigation (imagine moving around your house) compared to rest elicited significant activity in all the studied ROIs (i.e. pre-SMA, dorsal premotor cortex, parahippocampal cortex, retrosplenial cortex, occipito-parietal junction, precuneus) and for most of the healthy volunteers (see Table 2) at both 3T and 1.5T (see Table 3 and Figure 3). Group activations are shown in Table 4 and Figure 4.

**Table 3:** Individual results for the spatial navigation task.

Subject Number	1	2	3	4	5	6	7	8	9	10	11	12	13	14	Total	Peak Coordinates
																(x,y,z; mean ± SD)
*1.5T (1 run)*																
Pre-SMA	+	−	−	−	+	−	+	−	+	+	+	−	+	+	8	−3±4, 13±6, 58±5
																0±5, 13±5, 56±6
Dorsal premotor cortex	+	−	−	+	−	−	+	+	+	+	+	−	+	+	9	−35±6, 0±4, 54±2
																33±4, 3±5, 57±4
Parahippocampal cortex	−	+	−	−	+	+	+	−	+	+	−	−	+	−	7	−20±18, −32±6, −24±4
																28±4, −32±4, −28±4
Retrosplenial cortex	+	+	−	−	+	−	+	−	+	−	+	−	+	+	8	−14±3, −55±2, 6±4
																15±3, −53±4, 9±2
Occipito-parietal junction	+	+	−	−	+	−	+	−	+	+	+	−	+	+	9	−28±3, −73±7, 26±4
																26±4, −64±5, 18±4
Precuneus	+	+	−	−	+	−	+	+	+	+	+	−	+	+	10	−19±6, −72±4, 48±4
																20±3, −71±5, 48±4
**Total**	5	4	0	1	5	1	6	2	6	5	5	0	6	5		
*1.5T (2 runs)*																
Pre-SMA	+	−	+	+	+	+	+	−	+	−	+	−	+	+	10	−3±3, 11±3, 55±4
																0±3, 12±3, 54±4
Dorsal premotor cortex	+	+	+	+	+	+	+	−	+	−	+	−	+	+	11	−32±5, 0±3, 56±4
																38±6, 1±4, 56±2
Parahippocampal cortex	+	+	−	−	−	+	+	−	+	+	−	−	+	+	8	−28±1, −33±4, −21±2
																28±4, −30±6, −29±5
Retrosplenial cortex	+	+	+	−	+	+	+	−	+	−	+	−	+	+	10	−14±3, −56±3, 7±4
																15±3, −53±4, 9±2
Occipito-parietal junction	+	+	+	+	+	+	+	+	+	−	+	−	+	+	12	−28±5, −75±7, 28±3
																27±5, −67±5, 19±4
Precuneus	+	+	+	+	+	+	+	+	+	+	+	+	+	+	14	−19±6, −72±4, 47±3
																20±4, −73±5, 48±3
**Total**	6	5	5	4	5	6	6	2	6	2	5	1	6	6		
*3T*																
Pre-SMA	+	+	+	+	+	+	+	−	+	+	−	+	+	+	12	−1±4, 13±4, 54±3
																0±3, 13±4, 54±3
Dorsal premotor cortex	+	+	+	+	+	+	+	+	−	+	+	+	+	+	13	−33±7, 0±3, 53±4
																34±3, 3±4, 60±4
Parahippocampal cortex	−	+	−	+	+	+	−	−	−	−	+	−	+	+	7	−28±2, −37±2, −21±2
																31±2, −30±2, −25 ±0
Retrosplenial cortex	+	+	−	+	+	+	+	−	−	+	+	−	+	+	10	−12±3, −57±3, 8±3
																14±3, −54±4, 10±0
Occipito-parietal junction	+	+	−	+	+	+	+	−	−	+	+	−	+	+	10	−30±3, −77±5, 27±4
																30±5, −68±7, 20±4
Precuneus	+	+	−	+	+	+	+	−	+	+	+	+	+	+	12	−16±3, −72±4, 49±3
																20±4, −71±4, 49±3
**Total**	5	6	2	6	6	6	5	1	2	5	5	3	6	6		

Results thresholded at FWE corrected p<0.05 on the studied ROIs.

**Table 4:** Group results for the spatial navigation task.

Brain area	x	y	z	Z value	p value
*1.5 T (1 run)*					
Pre-SMA	−3	12	50	3.66	0.003
Dorsal premotor cortex	−29	0	55	3.84	0.001
	31	4	55	2.99	0.018
Parahippocampal cortex	−26	−37	−20	3.20	0.010
	31	−33	−25	3.35	0.006
Retrosplenial cortex	−14	−56	10	3.35	0.006
	16	−52	10	2.82	0.021
Occipito-parietal junction	−29	−82	30	2.93	0.020
	23	−60	15	3.35	0.006
Precuneus	−26	−67	45	4.16	0.000
	20	−67	50	3.06	0.014
*1.5 T (2 runs)*					
Pre-SMA	−3	12	50	3.84	0.001
Dorsal premotor cortex	−29	0	55	4.75	0.000
	31	0	65	3.53	0.004
Parahippocampal cortex	−22	−37	−25	4.30	0.000
	27	−37	−30	4.36	0.000
Retrosplenial cortex	−14	−56	10	3.45	0.005
	16	−52	10	3.37	0.005
Occipito-parietal junction	−26	−67	20	3.94	0.001
	23	−60	15	3.49	0.004
Precuneus	−26	−67	45	4.74	0.000
	20	−67	50	4.00	0.001
*3T*					
Pre-SMA	4	12	55	4.13	0.001
Dorsal premotor cortex	−29	0	55	4.48	0.000
	31	−3	60	4.04	0.001
Parahippocampal cortex	−26	−33	−20	3.52	0.004
	27	−30	−25	3.12	0.012
Retrosplenial cortex	−14	−56	10	3.96	0.001
	16	−48	10	4.36	0.000
Occipito-parietal junction	−29	−74	35	3.33	0.007
	23	−60	15	3.04	0.015
Precuneus	−26	−74	40	3.41	0.005
	20	−67	50	3.23	0.010

Regions showing significant activation versus rest for the 1.5T and 3T data. Results thresholded at FWE-corrected p<0.05.

### 1.5T vs 3T

At a group level, the participants showed stronger activity in SMA for motor imagery at 3T when compared to both 1 or 2 runs at 1.5T (Table 5). However, a repeated measures ANOVA on the individual subject results showed no effect of the magnetic field strength/number of runs (i.e. 1 run at 1.5T, 2 runs at 1.5T, 1 run at 3T) on the number of ROIs that showed significant activity per subject (F = 0.278, p>0.05).

**Table 5:** Group comparisons between 1.5T and 3T data for the motor imagery (tennis) task.

Brain area	x	y	z	Z value	p value
*1.5 T (1 run) vs 3 T*					
Pre-SMA	8	0	55	2.78	0.031
SMA	1	−3	55	3.17	0.012
*1.5 T (2 runs) vs 3 T*					
SMA	1	−7	55	3.03	0.016
Dorsal premotor cortex	34	−7	45	3.00	0.019

Regions showing significant differences between 1.5T and 3T data. Results thresholded at FWE-corrected p<0.05.

For spatial navigation the parahippocampal cortex, along with the occipito-parietal junction, showed stronger activity at 3T when compared to 1 run at 1.5T at a group level (Table 6). When two runs were considered at 1.5T, the occipito-parietal junction and precuneus showed stronger activity at 3T. However, a repeated measures ANOVA on the individual subject results showed no effect of the magnetic field strength/number of runs (i.e. 1 run at 1.5T, 2 runs at 1.5T, 1 run at 3T) on the number of ROIs that showed significant activity per subject (F = 1.890 p>0.05).

**Table 6:** Group comparisons between 1.5T and 3T data for the spatial navigation task.

Brain area	x	y	z	Z value	p value
*1.5 T (1 run) vs 3 T*					
Parahippocampal cortex	20	−82	45	4.25	0.000
Occipito-parietal junction	−22	−82	30	3.31	0.008
	31	−74	10	3.74	0.002
*1.5 T (2 runs) vs 3 T*					
Occipito-parietal junction	−26	−82	20	2.65	0.041
	31	−74	10	3.49	0.004
Precuneus	−18	−82	40	2.78	0.030
	23	−82	40	4.18	0.000

Regions showing significant differences between 1.5T and 3T data. Results thresholded at FWE-corrected p<0.05.

## Discussion

Mental imagery fMRI tasks have proven to be a successful approach to detecting command following in behaviorally non-responsive patients when 3T MRI scanners are used [4], [9]. However, these scanners are not widely available in clinical settings, which limits availability for most patients. Crucially, here we showed that brain activation previously described at 3T can be reliably and robustly replicated at a field strength of 1.5T,using a standard hospital scanner. To our knowledge, this is the first study to assess the single subject reliability of mental imagery fMRI tasks at these two magnetic field strengths.

A number of 3T studies have identified brain activity in SMA, pre-SMA and dorsal premotor cortex for motor imagery (i.e. imagine playing tennis) and pre-SMA, dorsal premotor cortex, parahippocampal cortex, retrosplenial cortex, occipito-parietal junction and precuneus for spatial navigation (i.e. imagine walking around your house) [4], [5], [9]. Our group findings at 3T are consistent with these results. More importantly, we were able to generate similarly robust activity in all of these areas when the participants were scanned in the clinical 1.5T MRI scanner. These results are consistent with previous evidence suggesting that it is possible to detect brain activity at 1.5T during mental imagery tasks [13]–[16].

When group data was directly compared between the two field strengths we found stronger activity at 3T in the SMA and the occipito-parietal junction for motor imagery and spatial navigation respectively, when compared to either 1 or 2 runs at 1.5T. Other areas, such as the pre-SMA, dorsal premotor cortex, parahippocampal cortex or precuneus, however, showed different effects depending on the number of runs included. The majority of previous studies comparing 1.5T and 3T data directly have been concerned with the reliability of structural MRI sequences. These investigations have typically demonstrated a better sensitivity of 3T scanners for detecting pathological changes such as those characteristic of tumours, neurological syndromes, or coronary diseases, amongst others [11], [17]–[20]. Although fMRI studies had not addressed such a comparison in clinical contexts, a number of methodological reports showed higher BOLD sensitivity at a field of 3T as compared to 1.5T in areas such as sensoriomotor cortices (e.g. SMA) [21], [22] or the parahippocampal cortex [23], both known to be engaged in the mental imagery tasks here studied. While our group results seem to be in agreement with the above studies, it is important to note that the differences were limited to small clusters in a minority of the regions studied.

In a study comparing several imagery tasks, Boly and colleagues [5] demonstrated that a motor imagery and spatial navigation produced the most consistent task-specific patterns of activation. Our results at the lower field of 1.5T further exemplify the reliability of these two tasks. Their real clinical applicability, however, lies in their use as a method for assessing the presence of volitional activity, and thus of awareness, in single non communicative brain-injured patients [5]. Indeed, previous use of these tasks has revealed a subset of patients who are aware but entirely physically unresponsive; thus, although they fulfil all the internationally agreed criteria for the vegetative state, which are based on behavioural signs, clear signs of command-following can be demonstrated using neuroimaging (see [2] for a review).

Consistent with previous reports at 3T[4], [5], [9], [24], we identified single subject activity in SMA, pre-SMA, dorsal premotor cortex and, although less consistently, inferior parietal lobule for motor imagery. At 1.5T we were able to obtain comparable results, even when just 1 run was included in the analysis. Similarly, for spatial navigation we found task-specific activity fully consistent with previous reports at 3T [4], [5], [9] with both the 3T and the 1.5T data, and independently of the number of runs. Furthermore, subsequent statistical analyses demonstrated that the field strength had no impact in the number or regions that showed positive results for each participant in either task. These results suggest that it is possible to use a paradigm including motor imagery and spatial navigation to robustly elicit covert command-following (and therefore detecting awareness) using a clinical 1.5T scanner. Taking into account the broader availability of 1.5T scanners in clinical settings over the world, our results pave the road for a more generalised use of these fMRI methods to ensure patients receive a diagnosis that adequately describes their cognitive abilities. To this end, although for this study we used equipment that was specifically designed to deliver stimuli in fMRI studies, the simplicity of this paradigm allows for a much simpler set up where task instructions and cues (i.e. ‘tennis’/‘house’ versus ‘relax’) could be directly delivered by the scanner operator using the patient intercom system.

While we were able to detect consistent task-specific activity for most participants, a small minority of them (n = 3) failed to show brain activity in some of their sessions or for one of the two tasks. Our paradigm was designed to minimise the possibility of false positives (that is, detecting awareness when the patient is not in fact aware) and several methods have been used to ensure that the signature patterns of activity only occur when the participants wilfully (that is, intentionally) follow the commands [25]. By contrast, no conclusions or claims about the preservation or loss of residual awareness in patients can be drawn on the basis of a negative finding (for an in-depth discussion see [2]or [26]). The absence of a positive fMRI outcome for these 3 (aware) healthy participants emphasises the importance of only drawing conclusions on the basis of positive results in patients, as these healthy participants show unequivocally that a null fMRI outcome does not necessarily indicate an absence of awareness. This result also stresses the need for repeated testing before strong conclusions can be made. Only one participant (C08) failed to show any positive results for the motor imagery task. In contrast, for the spatial navigation task, the two participants who failed to show a positive result at 1.5 in the spatial navigation task when only 1 run was considered (C03 and C12), were able to show significant activity with two runs.

On a related note, we found certain heterogeneity across participants in the number of regions engaged by each task. This suggests that patient results can be interpreted with certain flexibility and that finding activity in each and all the regions that are found at a group level should not be a requirement to consider the result as a positive and conclude a patient is aware. This is particularly relevant if we take into account that the brain damage that leads to a vegetative state and related disorders of consciousness is typically severe and widespread [27], [28], and focal lesions or post-injury brain reorganisation may affect the recruitment of particular areas during the performance of these (or any other) cognitive tasks. Monti and colleagues [9], for example, based their analyses on SMA and the parahippocampal area (for motor imagery and spatial navigation respectively). Because of the strong task-specificity that characterises these two regions, they were able to successfully use them as localisers to establish ‘yes’/‘no’ communication with a non-responsive patient. In contrast, in a more recent study [2], a vegetative state patient failed to engage the parahippocampal area in the spatial navigation task, but was able to consistently engage the occipito-parietal junction on a number of occasions and across multiple scan runs. This activity was then adopted as an indicator of command-following and, together with his activity in the SMA, it was used to successfully obtain ‘yes’/‘no’ answers to a number of autobiographical and clinically relevant questions [2]. Our results suggest that a similar approach that is based on the specific activity patterns elicited in each patient could be performed at 1.5T.

## Conclusions

Our results demonstrate that it is possible to use a paradigm including motor imagery and spatial navigation to elicit covert command-following, and therefore detect awareness, in clinical 1.5T MRI scanners. Moreover, the paradigm's reliability is comparable to that previously shown for 3T scanners in research settings. Thus, Our findings justify a broader use of mental imagery fMRI paradigms, which will make them available to a larger number of patients to ensure they receive an accurate diagnosis. We have shown that these fMRI tasks are feasible at 1.5T and propose that this could facilitate larger multi-centered studies to further test clinically relevant questions related to sensitivity, specificity or the prevalence of covert awareness in non-responsive patients. Importantly, this may also open the door for the assessment of potential covert awareness in new groups of patients that typically do not have access to research specialised MRI scanners, such as, those in a comatose state in the acute phases of the injury.
